# Diabetes Primes Neutrophils for Neutrophil Extracellular Trap Formation through Trained Immunity

**DOI:** 10.34133/research.0365

**Published:** 2024-04-23

**Authors:** Sanjeeb Shrestha, Yu-Bin Lee, Hoyul Lee, Yeon-Kyung Choi, Bo-Yoon Park, Mi-Jin Kim, Young-Jin Youn, Sun-Hwa Kim, Soo-Jung Jung, Dong-Keun Song, Hee Kyung Jin, Jae-Sung Bae, In-Kyu Lee, Jae-Han Jeon, Chang-Won Hong

**Affiliations:** ^1^ Department of Physiology, School of Medicine, Kyungpook National University, Daegu 41944, Republic of Korea.; ^2^ Leading-edge Research Center for Drug Discovery and Development for Diabetes and Metabolic Disease, Kyungpook National University Hospital, Daegu 41404, Republic of Korea.; ^3^ Research Institute of Aging and Metabolism, Kyungpook National University, Daegu 41404, Republic of Korea.; ^4^ Department of Internal Medicine, School of Medicine, Kyungpook National University, Kyungpook National University Chilgok Hospital, Daegu 41404, Republic of Korea.; ^5^ Department of Pharmacology, College of Medicine, Hallym University, Chuncheon 24252, Republic of Korea.; ^6^ Department of Laboratory Animal Medicine, College of Veterinary Medicine, Kyungpook National University, Daegu 41566, Republic of Korea.; ^7^ KNU Alzheimer’s disease Research Institute, Kyungpook National University, Daegu 41566, Republic of Korea.; ^8^ Department of Internal Medicine, School of Medicine, Kyungpook National University, Kyungpook National University Hospital, Daegu 41940, Republic of Korea.

## Abstract

Neutrophils are primed for neutrophil extracellular trap (NET) formation during diabetes, and excessive NET formation from primed neutrophils compromises wound healing in patients with diabetes. Here, we demonstrate that trained immunity mediates diabetes-induced NET priming in neutrophils. Under diabetic conditions, neutrophils exhibit robust metabolic reprogramming comprising enhanced glycolysis via the pentose phosphate pathway and fatty acid oxidation, which result in the accumulation of acetyl-coenzyme A. Adenosine 5′-triphosphate-citrate lyase-mediated accumulation of acetyl-coenzyme A and histone acetyltransferases further induce the acetylation of lysine residues on histone 3 (AcH3K9, AcH3K14, and AcH3K27) and histone 4 (AcH4K8). The pharmacological inhibition of adenosine 5′-triphosphate-citrate lyase and histone acetyltransferases completely inhibited high-glucose-induced NET priming. The trained immunity of neutrophils was further confirmed in neutrophils isolated from patients with diabetes. Our findings suggest that trained immunity mediates functional changes in neutrophils in diabetic environments, and targeting neutrophil-trained immunity may be a potential therapeutic target for controlling inflammatory complications of diabetes.

## Introduction

Activated innate immune cells exhibit long-term functional reprogramming, characterized by metabolic reprogramming and epigenetic modifications in response to specific immunological environments [1]. This process, known as trained immunity, drives the nonspecific memory of innate immune cells, ensuring a robust inflammatory response against subsequent stimulations. However, exaggerated responses by trained immunity can lead to detrimental inflammation [1]. In diabetes, innate immune cells undergo trained immunity due to the metabolic disturbances. Diabetic macrophages exhibit metabolic reprogramming and epigenetic modifications for proinflammatory genes [2,3]. This trained immunity results in a proinflammatory phenotype polarization of macrophages, thereby contributing to the chronic inflammatory status in patients with diabetes [3,4].

Neutrophils, the most abundant innate immune cells in humans, exhibit dysregulated functions in diabetes. Most effector functions of neutrophils, such as the generation of reactive oxygen species (ROS) generation, degranulation, chemotaxis, and phagocytosis, are dysregulated in diabetes [5,6]. Diabetic neutrophils (DNs) exhibit enhanced neutrophil extracellular trap (NET) formation [7,8]. Components of NETs, such as double-stranded DNA and granules, are found in the serum of patients with type 2 diabetes mellitus [7,9,10]. In addition, the basal levels of NETs are elevated in neutrophils isolated from these patients [9]. Moreover, neutrophils isolated from diabetic patients exhibit increased expression levels of peptidyl arginine deiminase 4, a key enzyme required for chromatin decondensation during NET formation [8]. These primed neutrophils are readily activated in response to inflammatory challenge. Consequently, NETs released by these primed neutrophils compromise wound healing in diabetic patients [7,11]. While these findings strongly suggest the trained immunity of neutrophils during diabetes, neither metabolic reprogramming nor epigenetic changes in neutrophils in diabetes have been elucidated so far.

In this study, we demonstrate that trained immunity mediates priming of neutrophils for NET formation in diabetes. Under diabetic conditions, neutrophils exhibit metabolic reprogramming, characterized by enhanced glycolysis, activation of pentose phosphate pathway (PPP), and increased fatty acid oxidation (FAO). Adenosine 5′-triphosphate (ATP)-citrate lyase (ACLY), a nuclear-cytosolic enzyme responsible for converting citrate to acetyl-coenzyme A (CoA), facilitates the accumulation of acetyl-CoA in the nucleus. Histone acetyltransferases (HATs) further induce histone acetylation, a prerequisite for the chromatin decondensation required for NET priming in diabetes. Inhibition of ACLY reverses delayed wound healing in a murine model of diabetes. These results advance our understanding of how trained immunity mediates the dysregulation of neutrophils in diabetes.

## Results

### Diabetes induces metabolic reprogramming in neutrophils

**Fig. 1.**
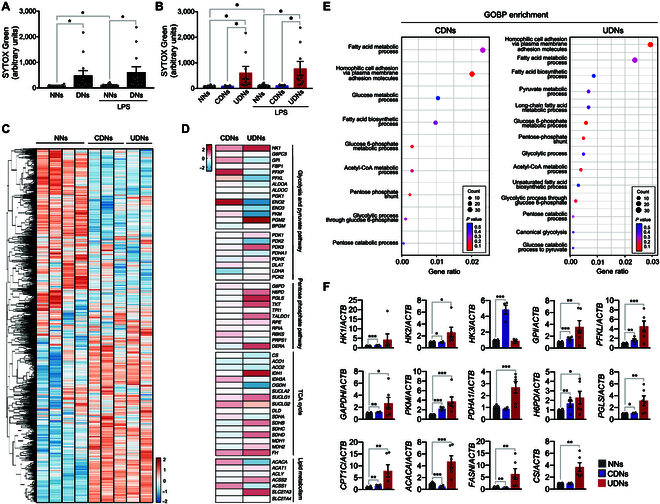
Metabolic reprogramming in neutrophils isolated from patients with diabetes. (A and B) Priming for NET formation in neutrophils isolated from patients with diabetes. Neutrophils were isolated from patients with diabetes (DNs) or healthy volunteers (NNs) in the presence or absence of LPS (10 μg/ml), and NET formation was analyzed using SYTOX Green staining. CDNs, neutrophils isolated from diabetic patients with controlled levels of HbA1c in serum; UDNs, neutrophils isolated from diabetic patients with uncontrolled levels of HbA1c. *n* = 12 per group. (C to E) RNA sequencing analysis of NNs (*n* = 4) and DNs (CDNs, *n* = 3; UDNs, *n* = 2). (C) Heatmap representations of DEGs in NNs and DNs. (D) Heatmap depicting DEG analysis of metabolic profiles of DNs with respect to NNs. (E) GO enrichment analysis of up-regulated genes in DNs. (F) A qPCR validation of enzymatic genes of metabolic pathways in DNs and NNs. NNs, *n* = 12; CDNs, *n* = 4; UDNs, *n* = 6. All results are expressed as means ± SEM. *P < 0.05; **P < 0.01; ***P < 0.001.

First, we confirmed the NET priming in neutrophils in diabetes. The demographic and clinical characteristics of patients with diabetes are summarized in Table S1. Neutrophils isolated from patients with diabetes (DNs) exhibited enhanced basal NET formation and showed an increased NET formation in response to subsequent lipopolysaccharide (LPS; 10 μg/ml) stimulation compared to neutrophils isolated from healthy volunteers [normal-glucose-exposed neutrophils (NNs)] (Fig. 1A). To evaluate the effects of long-term exposure to high-glucose condition on neutrophils, we categorized DNs according to their adjusted hemoglobin A1c (HbA1c) levels, based on blood glucose levels [12]. Neutrophils isolated from diabetic patients with uncontrolled levels of HbA1c [uncontrolled DNs (UDNs)] exhibited substantially higher basal levels of NET formation and demonstrated enhanced NET formation in response to LPS stimulation than those isolated from either diabetic patients with controlled HbA1c [controlled DNs (CDNs)] or NNs (Fig. 1B).

To investigate metabolic reprogramming in DNs, we conducted transcriptome analysis using RNA sequencing of NNs (*n* = 4) and DNs (CDNs, *n* = 3; UDNs, *n* = 2). We found 5,120 (2,462 up-regulated) and 2,490 (1,642 up-regulated) differentially expressed genes (DEGs) in CDNs and UDNs, respectively (Fig. 1C and Table S2). DEG analysis of the metabolic profiles of DNs with respect to NNs revealed increased expressions of key enzymes in metabolic pathways such as hexokinase 1 (*HK1*), phosphoglucomutase 2 (*PGM2*), hexose-6-phosphate dehydrogenase (*H6PD*), and 6-phosphogluconolactonase (*PGLS*) in UDNs (Fig. 1D and Fig. S1A). Gene Ontology (GO) enrichment analysis and Kyoto Encyclopedia of Genes and Genomes (KEGG) enrichment analysis revealed the up-regulation of the PPP in UDNs (Fig. 1E, Fig. S1B to E, and Table S2). We further examined the rate-limiting enzymes involved in metabolic pathways using quantitative polymerase chain reaction (qPCR). UDNs exhibited increased expression of genes involved in glucose metabolism [*HK2*, glucose-6-phosphate isomerase (*GPI*), 6-phosphofructokinase liver type (*PFKL*), glyceraldehyde-3-phosphate dehydrogenase (*GAPDH*), and pyruvate kinase muscle isozyme (*PKM*)], PPP (*H6PD* and *PGLS*), pyruvate metabolism [pyruvate dehydrogenase (PDH) E1 subunit α_1_ (*PDHA1*)], fatty acid metabolism [carnitine palmitoyltransferase 1C (*CPT1C*), fatty acid synthase (*FASN*), and acetyl-CoA carboxylase α (*ACACA*)], and the conversion of acetyl-CoA into citrate [citrate synthase (*CS*)] than NNs (Fig. 1F and Fig. S1F). CDNs exhibited alterations in metabolic genes to a lesser extent than did UDNs (*HK1*, *GPI*, *PFKL*, *GAPDH*, *PKM*, *H6PD*, and *CPT1C*) or down-regulation (*HK2*, *PGLS*, *FASN*, and *ACACA*) (Fig. 1F and Fig. S1F). No substantial changes were found in the expression levels of genes involved in the tricarboxylic acid (TCA) cycle in the DNs.

### High-glucose condition induces metabolic reprogramming in neutrophils

**Fig. 2.**
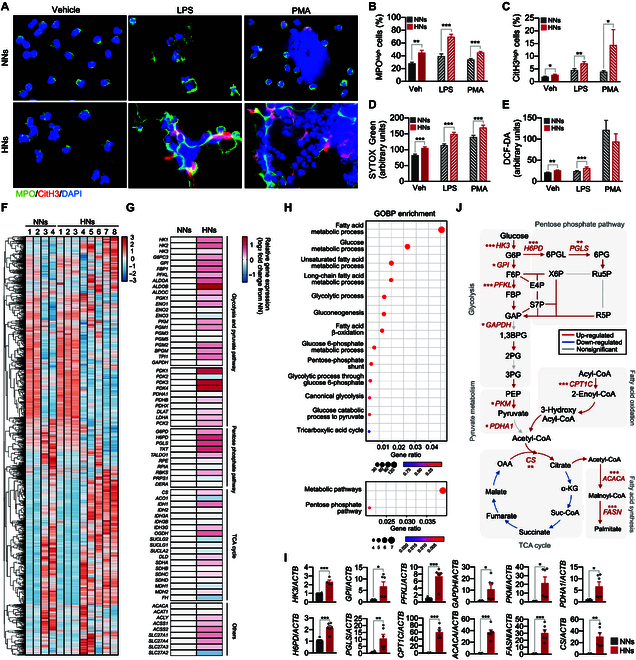
Metabolic pathways involved in high-glucose-induced NET priming. (A to E) The effect of high-glucose condition on the function of neutrophils. Human neutrophils were incubated in an RPMI medium supplemented with either 5.5 mM glucose (NNs) or 22 mM glucose (HNs) for 4 h. NNs and HNs were stimulated with LPS (10 μg/ml) or PMA (1 μg/ml) for 1 h. *n* = 4 to 7 per group. (A) Immunofluorescence images of NET formation in NNs and HNs. Representative images of 5 experiments are shown. Red, CitH3; green, MPO; blue, DAPI. (B and C) Percentages of MPO^high^ (B) and CitH3^high^ (C) neutrophils. (D) NET formation in NNs and HNs was determined by SYTOX Green staining. *n* = 7 per group. (E) ROS generation in NNs and HNs was determined by DCF-DA staining. *n* = 5 per group. (F to J*)* RNA sequencing analysis of NNs and HNs. *n* = 4 per group. (F) Heatmap representation of DEGs in NNs and HNs. (G) Heatmap depicting DEG analysis of metabolic profiles in NNs and HNs. (H) GO enrichment analysis of up-regulated genes in HNs. (I) A qPCR validation of the enzymatic genes of metabolic pathways in NNs and HNs. *n* = 6 per group. (J) Schematic depicting altered metabolic pathways in HNs based on KEGG metabolic pathway mapping and qPCR. Adjusted *P* < 0.05 was considered to indicate statistical significance for KEGG metabolic pathway mapping (colored arrows). The significant alterations in genes involved in metabolic pathways on qPCR analysis were denoted by asterisks. All results are expressed as means ± SEM. *P < 0.05; **P < 0.01; ***P < 0.001.

As our results suggested that prolonged exposure to high glucose during diabetes induced NET priming and metabolic reprogramming in neutrophils, we next examined the effects of high glucose on the functions of neutrophils. Neutrophils were incubated in RPMI medium containing either 5.5 mM glucose (NNs) or 22 mM glucose [high-glucose-exposed neutrophils (HNs)] for 4 h. Then, the neutrophils were stimulated with either LPS (10 μg/ml) or phorbol 12-myristate 13-acetate (PMA; 1 μg/ml) for 1 h, and NET formation was analyzed. HNs exhibited higher basal NET formation and higher NET formation in response to LPS and PMA stimulation than NNs (Fig. 2A to E). HNs also showed a remarkable enhancement in both basal- and LPS-induced ROS production (Fig. 2E). Neutrophils exposed to 11 mM glucose exhibited increased NET formation and ROS generation, and replacement with mannitol, nonmetabolizable sugar alcohol, at concentrations of either 11 or 22 mM, did not affect either basal NET formation or ROS generation, suggesting that high glucose per se is responsible for NET priming (Fig. S2A and B).

To further confirm the metabolic modulation of neutrophils under high-glucose conditions, we characterized the metabolic profile of HNs (*n* = 8) with respect to NNs (*n* = 4) using transcriptome data. The gene expression profiles of NNs and HNs were distinct and revealed a total of 6,407 (6,366 up-regulated) DEGs in HNs (Fig. 2F and Table S2). Similar to DNs, HNs exhibited the up-regulated genes involved in glycolysis, PPP, and pyruvate metabolism (Fig. 2G). Gene enrichment analysis of up-regulated genes for GO biological process (GOBP) and KEGG pathways showed enrichment of genes involved in glycolysis and PPP in HNs (Fig. 2H, Fig. S1C and D, and Table S2). We further confirmed the changes in the rate-limiting enzymes involved in metabolic pathways using qPCR. HNs showed increased expressions of genes involved in glucose metabolism (*HK3*, *GPI*, *PFKL*, *GAPDH*, and *PKM*), PPP (*H6PD*, *PGLS*), pyruvate metabolism (*PDHA1*), fatty acid metabolism (*CPT1C*, *ACACA*, and *FASN*), and conversion of acetyl-CoA into citrate (*CS*) (Fig. 2I and J and Fig. S2E to J). Neutrophils exposed to 11 mM glucose also demonstrated notable up-regulation of key metabolic genes, although this was less pronounced compared to exposure to 22 mM glucose (Fig. S2K).

### Metabolic reprogramming controls NET priming under high glucose

**Fig. 3.**
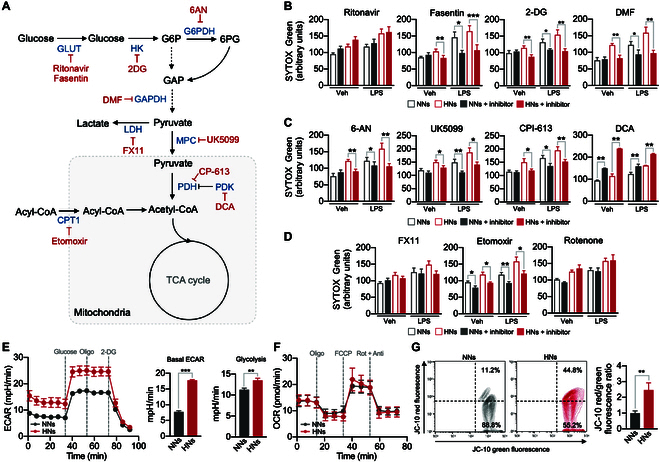
Metabolic modulation mediates NET priming. (A) Schematic depicting the inhibitors of the metabolic pathways. (B to D) The effects of inhibitors on metabolic pathways in high-glucose-induced NET priming. Neutrophils were incubated with either 5.5 mM glucose medium (NNs) or 22 mM glucose medium (HNs) in the presence or absence of the indicated inhibitors of metabolic pathways and then stimulated with LPS (10 μg/ml). The NET formation was analyzed using SYTOX Green staining. *n* = 5 to 7 per group. (E) Quantification of ECARs in NNs and HNs. Oligo, oligomycin. *n* = 15 per group. (F) Quantification of OCRs in NNs and HNs. Rot, rotenone; Anti, antimycin A. *n* = 12 per group. (G) Representative flow cytometry plots of the membrane potential of neutrophils as measured using JC-10 fluorescence. Red, JC-10 aggregates; green, JC-10 monomers. The bar graph shows the ratios of JC-10 aggregate and JC-10 monomer expression in neutrophils. *n* = 3 per group. All results are expressed as means ± SEM. *P < 0.05; **P < 0.01; ***P < 0.001.

As the transcriptome profile showed alterations in glucose utilization in neutrophils under diabetic conditions, we first examined the effects of inhibitors on glucose transporters during high-glucose-induced NET priming. Neutrophils were incubated for 4 h under either normal or high-glucose conditions in the presence or absence of glucose transporter inhibitors and further stimulated with LPS (10 μg/ml; Fig. 3A). Ritonavir, an inhibitor of glucose transporter 1 (GLUT1), did not affect high-glucose-induced NET priming, whereas fasentin, an inhibitor of GLUT1 and GLUT4, significantly inhibited basal NET formation and priming (Fig. 3B). These results suggest that neutrophils primarily utilize GLUT4 over GLUT1 for glucose uptake. Therefore, we examined the presence of GLUTs in neutrophils. NNs expressed GLUT1, GLUT3, and GLUT4 (Fig. S3A), consistent with the findings of a previous study [13]. Notably, HNs exhibited increased GLUT4 expression (Fig. S3A) and higher glucose uptake (Fig. S3B) than those of NNs. These results suggest that neutrophils actively utilize glucose under high-glucose conditions, and we further examined the metabolic pathways involved in high-glucose-induced NET priming.

Neutrophils were exposed to different glucose concentrations in the presence of various inhibitors of metabolic pathways, and priming for NET formation was examined. Inhibitors of the glycolytic pathway [2-deoxyglucose (2-DG), a hexokinase inhibitor; *N,N*-dimethylformamide (DMF), a GAPDH inhibitor] completely inhibited the high-glucose-induced NET priming (Fig. 3B). The inhibition of the PPP with 6-aminonicotinamide [6-AN; an inhibitor of glucose-6-phosphate dehydrogenase (G6PD)] completely inhibited the high-glucose-induced NET priming (Fig. 3C). Inhibition of pyruvate metabolism (with UK5099, a mitochondrial pyruvate transporter inhibitor, and CPI-613, a PDH inhibitor) also inhibited high-glucose-induced NET priming, whereas treatment with dichloroacetic acid (DCA, a pan-PDH kinase inhibitor) enhanced high-glucose-induced NET priming (Fig. 3C). In contrast, inhibition of lactate dehydrogenase A (LDHA) with FX11 did not affect the basal NET formation or high-glucose-induced NET priming (Fig. 3D). These results suggest that HNs utilize the glycolytic pathway by passing through the PPP for NET priming. Inhibition of FAO with etomoxir, an inhibitor of CPT1, inhibited the high-glucose-induced NET priming, whereas rotenone, an inhibitor of mitochondrial respiratory chain complex I, did not affect NET priming (Fig. 3D). Moreover, diphenyleneiodonium chloride (DPI), an inhibitor of reduced form of nicotinamide adenine dinucleotide phosphate (NADPH) oxidase, completely inhibited high-glucose-induced NET priming, suggesting that ROS-dependent NET priming (Fig. S3E). To further examine whether neutrophils are also primed for inflammatory cytokine production during diabetes, we measured the protein levels of tumor necrosis factor-α, interleukin-8 (IL-8), and IL-1β in neutrophils. HNs exhibited the increased levels of IL-8 and IL-1β in resting state, and LPS stimulation further enhanced the production of IL-1β in HNs than NNs (Fig. S3F).

We further explored the bioenergetics of HNs. Analysis of the extracellular acidification rate (ECAR), an indicator of aerobic glycolysis, revealed higher glycolysis rates in HNs than in NNs (Fig. 3E and Fig. S3C). The addition of oligomycin, an inhibitor of ATP synthase, did not affect ECAR in either HNs or NNs, suggesting a minimal role of mitochondria in neutrophil energetics (Fig. 3E). In contrast, no substantial differences were found in the oxygen consumption rate (OCR), an indicator of mitochondrial oxidative phosphorylation, between HNs and NNs (Fig. 3F and Fig. S3C). However, analysis of mitochondrial transmembrane potential using MitoTracker and JC-10 revealed the existence of functional mitochondria in neutrophils (Fig. 3G and Fig. S3D). Moreover, HNs displayed greater MitoTracker fluorescence and greater JC-10 red/green fluorescence ratios than NNs (Fig. 3G and Fig. S3D). These results suggest that although mitochondrial respiration plays a minimal role, functional mitochondria are required for high-glucose-induced NET priming.

**Fig. 4.**
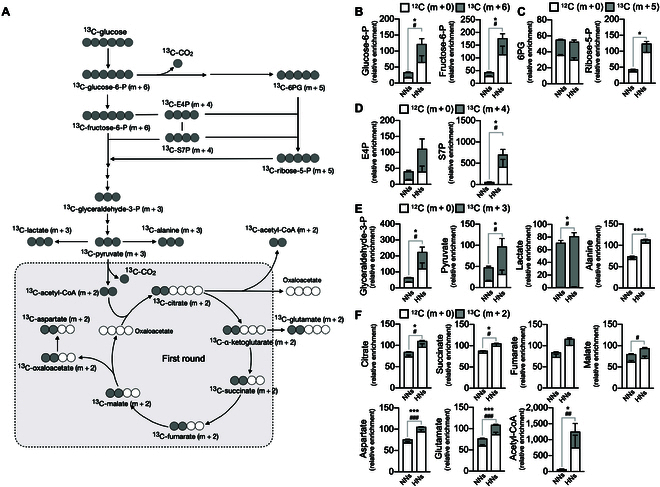
Validation of metabolic modulation in HNs through U-^13^C glucose tracing. Metabolic flux analysis in NNs and HNs. Neutrophils were incubated in a medium supplemented with U-^13^C glucose, and the fates of labeled carbon were traced using LC-mass spectrometry. The relative enrichment with respect to concentration of each metabolite from NNs was shown. (A) The schematic diagram for incorporation and distribution patterns of U-^13^C glucose into downstream metabolites associated with glycolysis, PPP, and TCA cycle. (B to F) Distribution of U- ^13^C (m + 6) and ^12^C (m + 0) into different metabolites. (B) U-^13^C (m + 6) in glucose-6-phosphate (glucose-6-P) and fructose-6-phosphate (fructose-6-P). (C) U-^13^C (m + 5) in 6-phosphogluconate (6-PG) and ribose-5-phosphate (ribose-5P). (D) U-^13^C (m + 4) in erythose-4-phosphate (E4P) and seduheptulose-7-phosphate (seduheptulose-7-P). (E) U-^13^C (m + 3) in glyceraldehyde-3-phosphate (glyceraldehyde-3-P), pyruvate, lactate, and alanine. (F) U-^13^C (m + 2) in citrate, succinate, fumarate, malate, aspartate, glutamate, and acetyl-CoA. *n* = 3 to 5 per group. Data are presented as relative metabolite abundance and expressed as means ± SEM. *P < 0.05; ***P < 0.001; #*P *< 0.05; ##*P *< 0.01; ###*P *< 0.001.

To trace the metabolic reprogramming of neutrophils under diabetic conditions, we examined changes in metabolic intermediates through unbiased metabolic flux analysis using ^13^C-U-glucose (Fig. 4A). The HNs exhibited significant enrichment of ^13^C-enriched intermediates in glycolysis, PPP, and pyruvate metabolism (Fig. 4B to E and Fig. S4A to C). Mitochondrial TCA cycle intermediates (m + 2), such as α-ketoglutarate, succinate, fumarate, and malate, were mostly unlabeled (Fig. 4F and Fig. S4D). Intriguingly, the amount of acetyl-CoA was drastically increased in HNs (Fig. 4D and Fig. S4E), indicating that glucose is hardly incorporated into the TCA cycle and intermediates of the TCA cycle are rapidly catalyzed into acetyl-CoA in HNs. Moreover, the total amount of unlabeled acetyl-CoA was also increased in HNs, supporting the notion that nonglucose sources, such as fatty acids, contribute to the accumulation of acetyl-CoA in HNs.

### Accumulation of acetyl-CoA mediates NET priming under high glucose

**Fig. 5.**
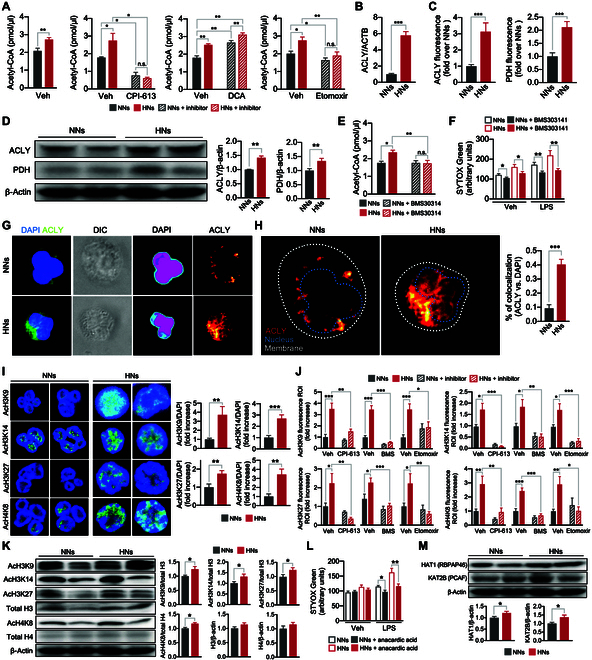
ACLY mediates histone acetylation that primes NET priming in HNs. (A) Accumulation of acetyl-CoA in neutrophils. Neutrophils were incubated in a normal- or high-glucose medium in the presence or absence of the indicated inhibitors, and the intracellular concentrations of acetyl-CoA were examined using enzyme-linked immunosorbent assay. *n* = 4 to 8 per group. (B) Relative ACLY mRNA levels in NNs and HNs. *n* = 6 per group. (C) Quantification of ACLY and PDH fluorescence in NNs and HNs. *n* = 16 per group. (D) Western blotting of total ACLY and PDH (α_1_ and α_2_ subunits) in NNs and HNs. The bar graph shows the fold changes in the expression levels of total ACLY and PDH compared with β-actin. *n* = 9 to 10 per group. (E and F) Effects of ACLY inhibition on high-glucose-induced (E) acetyl-CoA accumulation and (F) NET priming. *n* = 8 per group. (G) Representative images of the subcellular localization of ACLY in neutrophils. The subcellular localization of reporters was analyzed using an EzColocalization analysis. Left: Immunofluorescence images showing reporter 1 (blue, DAPI) and reporter 2 (green, ACLY). Second: Differential interference contrast (DIC) images of cell identification; Third: Heatmaps for DAPI. Right: Heatmaps for ACLY. The signal intensities are indicated by the bar on each reporter image. Representative images of 5 independent experiments are shown. (H) The probability of the colocalization of ACLY with the nucleus determined using PCC metric in the EzColocalization analysis. Representative images of 5 independent experiments are shown. The bar graph denotes the percentages of colocalization between ACLY and the nucleus. *n* = 5 per group. (I) Representative confocal images depicting the levels of acetylation of the indicated histones in neutrophils. The bar graph shows the fold changes in the expression levels of the indicated acetylated histones in HNs compared to NNs. *n* = 4 to 7 per group. (J) Representative western blotting images and bar graphs of histone acetylation in NNs and HNs. *n* = 6 per group. (K) Effects of inhibitors on histone acetylation in neutrophils. Neutrophils were incubated under either normal- or high-glucose conditions in the presence or absence of the indicated inhibitors. *n* = 6 to 8 per group. (L) Effects of histone acetylase inhibition on high-glucose-induced NET priming. Neutrophils were incubated under either normal- or high-glucose conditions in the presence or absence of anacardic acid. *n* = 13 per group. (M) Representative Western blotting images and bar graphs of the expression levels of HAT1 (RBAP46) and KAT2B (PCAF). *n* = 9 to 10 per group. CPI-613, an inhibitor of PDH; DCA, an inhibitor of PDH kinase; BMS303141, an inhibitor of ACLY; etomoxir, an inhibitor of CPT-1. All results are expressed as means ± SEM. *P < 0.05; **P < 0.01; ***P < 0.001.

We further confirmed the accumulation of acetyl-CoA in HNs using an endpoint fluorometric assay. HNs showed significantly higher intracellular concentrations of acetyl-CoA than NNs (Fig. 5A), which is consistent with the metabolic flux analysis results. We further confirmed the involvement of metabolic modulation in acetyl-CoA accumulation in HNs using inhibitors of key metabolic pathways. Inhibition of PDH using CPI-613 completely inhibited the accumulation of acetyl-CoA in HNs, whereas the activation of PDH using DCA further enhanced the accumulation of acetyl-CoA in HNs (Fig. 5A). Inhibition of FAO using etomoxir also inhibited the accumulation of acetyl-CoA in HNs (Fig. 5A).

These results suggested that metabolic reprogramming led to acetyl-CoA accumulation in DNs. DNs exhibited no substantial alterations in the expression levels of enzymes involved in the TCA cycle (Figs. 1D and 2G), and the expression of CS increased (Figs. 1F and 2I and J), suggesting that citrate might accumulate in these neutrophils. However, in line with our expectation, the concentration of citrate was not highly increased in HNs (Fig. 4F), and the acetyl-CoA accumulated in HNs to a greater level than anticipated (Figs. 4F and 5A). Therefore, we reasoned that an additional metabolic pathway was required for acetyl-CoA accumulation in DNs. ACLY is a nuclear-cytosolic enzyme that generates acetyl-CoA from citrate, which is produced through the mitochondrial TCA cycle [14]. Therefore, we determined the expression levels of ACLY in neutrophils. The results of qPCR (Fig. 5B) and immunofluorescence microscopic (Fig. 5C and Fig. S5A) analyses showed increased ACLY expression in HNs. Moreover, the results of the immunoblotting analysis revealed that ACLY expression was higher in HNs than in NNs (Fig. 5D). The expression of PDH, a key enzyme responsible for the oxidative decarboxylation of pyruvate into acetyl-CoA, was also significantly enhanced in HNs (Fig. 5C and D and Fig. S5B). To further demonstrate that ACLY is required for high-glucose-induced NET priming, we examined the effects of an ACLY inhibitor, BMS303141, in HNs. BMS303141 inhibited acetyl-CoA accumulation in HNs (Fig. 5E) and high-glucose-induced NET priming (Fig. 5F). We next hypothesized that acetyl-CoA accumulates in the nuclear compartment rather than the mitochondria, CS converts acetyl-CoA into citrate in the mitochondria, and ACLY converts citrate into acetyl-CoA again in the nucleus. HNs exhibited increased nuclear translocation of ACLY (Fig. 5G and H), whereas PDH did not (Fig. S5C).

Acetyl-CoA is a central metabolite for energy production through the TCA cycle and an important acetyl source for histone acetylation [14]. Recent studies have suggested that histone acetylation contributes to the NET formation through the decondensation of chromatin [15,16]. Furthermore, ACLY provides acetyl-CoA in the nucleus and facilitates histone acetylation in various cell types [14,17] including macrophages [18] and T helper 17 (T_H_17) cells [19]. Therefore, we hypothesized that the increased accumulation of acetyl-CoA contributes to histone acetylation in HNs, leading to the priming of neutrophils for NET formation. Immunofluorescence microscopic analysis revealed greater acetylation of lysine residues 9, 14, and 27 on histone 3 (AcH3K9, AcH3K14, and AcH3K27, respectively) and of lysine-8 on histone 4 (AcH4K8) in HNs than in NNs (Fig. 5I and Fig. S5D to G). Immunoblot analysis confirmed increased histone acetylation in HNs (Fig. 5K). To further explore correlations between metabolic modulation and histone acetylation, we investigated the effects of metabolic pathway inhibitors on histone acetylation in HNs. We found that CPI-613, BMS303141, and etomoxir completely inhibited the histone acetylation in HNs (Fig. 5K and Fig. S5D to G).

We next determined whether the increased acetylation of histones was responsible for NET priming under high glucose. Anacardic acid, a pan-inhibitor of HATs, completely inhibited the high-glucose-induced NET priming (Fig. 5L). We further determined the expression levels of HATs, HAT1 and K (lysine) acetyltransferase 2B (KAT2B), in neutrophils and found that expressions levels of HAT1 and KAT2B were increased in HNs (Fig. 5M).

### Diabetes primes neutrophils for NET formation through trained immunity

**Fig. 6.**
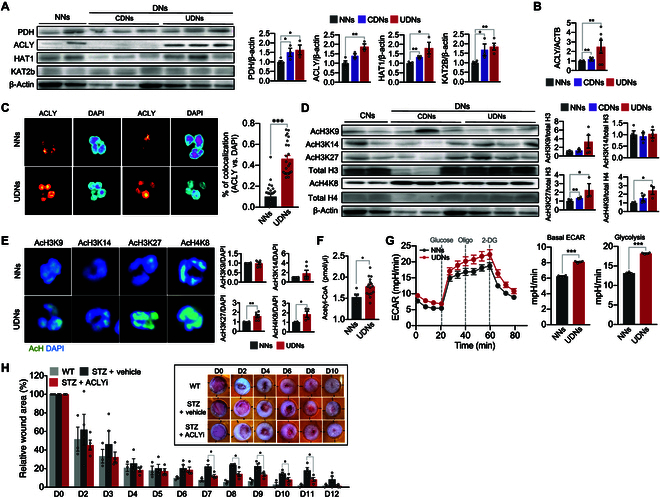
Diabetes primes NET formation via trained immunity. (A) Representative Western blotting images and bar graphs of the expression levels of PDH, ACLY, HAT1, and KAT2b in NNs and DNs. NNs, *n* = 4 to 5; CDNs, *n* = 3; UDNs, *n* = 4. (B) The expression levels of ACLY in NNs and DNs. NNs, *n* = 12; CDNs, *n* = 4; UDNs, *n* = 6. (C) Representative images of the subcellular localization of ACLY in DNs and CDNs using EzColocalization analysis. The bar graph denotes the probability of colocalization of ALCY with the nucleus in DNs and NNs using PCC metric. Each dot represents a single cell. Representative images of 3 independent experiments are shown. *n* = 3 per group. (D) Representative Western blotting images and bar graphs of the expression levels of acetylated histones in DNs and NNs. NNs, *n* = 4 to 6; CDNs, *n* = 4; UDNs, *n* = 3. (E) Representative confocal images depicting the levels of acetylation of the indicated histones in neutrophils. The bar graph shows the fold changes in the expression levels of the indicated acetylated histones in DNs compared to NNs. *n* = 2 to 3 per group. Each sample was analyzed in duplicates, resulting in 2 data point per sample. (F) The intracellular concentration of acetyl-CoA in NNs and DNs. NNs, *n* = 5; UDNs, *n* = 8. Each patient sample was analyzed in duplicates, resulting in 2 data point per patient. (G) Quantification of ECARs in DNs and NNs. *n* = 4 per group. (H) The effect of an inhibitor for ACLY on wound healing in a murine model of diabetes. The excisional dorsal full-thickness skin wounds (6 mm in diameter) were induced in the center of each dorsal skin of C57BL/6J mice. The wound closure results were quantified on days 1, 2, 4, 6, 8, and 10 after wounding. Inset: Representative photographs of wound healing. WT (wild-type control), *n* = 3; STZ + vehicle (vehicle-treated diabetic mice), *n* = 4; STZ + ACLYi (ACLY inhibitor-treated diabetic mice), *n* = 4. All results are expressed as means ± SEM. *P < 0.05; **P < 0.01; ***P < 0.001.

On the basis of the finding that high-glucose condition primed neutrophils for NET formation through trained immunity, we further examined the key molecules involved in metabolic reprogramming and epigenetic changes in DNs. The expression levels of PDH, ACLY, HAT1, and KAT2B were significantly increased in the UDNs (Fig. 6A and B). Moreover, UDNs exhibited the increased nuclear translocation of ACLY (Fig. 6C). Among the acetylated histones found in HNs, UDNs exhibited increased expressions of AcH3K27 and AcH4K8 (Fig. 6D and E). UDNs also showed increased accumulation of acetyl-CoA (Fig. 6F) and higher glycolysis rates than NNs (Fig. 6G). The serum concentrations of tumor necrosis factor-α, IL-8, and IL-1β were elevated in patients with uncontrolled levels of HbA1c, and UDNs showed increased basal levels of IL-1β and robust IL-1β production in response to LPS stimulation (Fig. S6A).

To validate whether neutrophils primed through trained immunity contribute to delayed wound healing in diabetes, we examined the effect of ACLY inhibitors on wound healing using a murine model of diabetes. Wound closure in streptozotocin (STZ)-induced diabetic mice was substantially delayed compared to that in control mice, and the treatment with an inhibitor of ACLY successfully reversed delayed wound closure (Fig. 6H and Fig. S6B to D).

## Discussion

In this study, we investigated the trained immunity of neutrophils under diabetic conditions. DNs exhibit metabolic reprogramming characterized by enhanced glycolysis, PPP, and FAO, resulting in the accumulation of acetyl-CoA. ACLY mediates the accumulation of acetyl-CoA, and HATs further mediate epigenetic modification through acetylation of histones, resulting in priming for NET formation. Activated neutrophils preferentially utilize glycolysis for effector functions, such as NET formation and ROS generation [20,21]. They exhibit increased uptake of glucose [13,21], and inhibition of the glycolytic pathway inhibits most effector functions of neutrophils, such as chemotaxis, NET formation, and ROS generation [20,21]. Consistent with these findings, HNs exhibited an increased GLUT4 expression with enhanced glucose uptake. Most genes involved in glycolytic pathways were up-regulated, and ECAR was substantially increased in DNs. Furthermore, pharmacological inhibition of the glycolytic pathway suppressed high-glucose-induced NET priming, and metabolic intermediates of glycolysis were also increased in HNs, suggesting the indispensable role of glycolysis in high-glucose-induced NET priming. DNs utilized glycolysis by passing through the PPP for NET priming. The levels of enzymes involved in the PPP were up-regulated in DNs, and the pharmacological inhibition of G6PD completely abrogated high-glucose-induced NET priming. Moreover, the metabolic intermediates of PPP were highly increased in HNs, and DPI completely inhibited high-glucose-induced NET priming. These results suggest that PPP is also indispensable for high-glucose-induced NET priming. PPP allows the diversion of the glycolytic pathway to generate NADPH [22], a key substrate for NADPH oxidase, during ROS generation in activated neutrophils. Neutrophils use the metabolic shift to PPP for amyloid- and PMA-induced NET formation [20]. However, pharmacological inhibition of the later phases of the glycolytic pathway, such as UK5099 and CPI-613, also completely inhibited high-glucose-induced NET priming, suggesting that glucose is redirected to PPP in the early phase of glycolysis to replenish NADPH oxidase and is then reincorporated into the glycolytic pathway.

We found that DNs utilize FAO for high-glucose-induced NET priming. Immature neutrophils utilize lipophagy-mediated FAO for energy production [23]. FAO mediates oxidized low-density-lipoprotein-induced NET formation in mature neutrophils [24], and a previous study has proposed a hypothesis regarding compensatory FAO utilization in neutrophils during hyperglycemia [25]. In the present study, the increased expression of FAO enzymes, such as CPT1C, ACACA, and hydroxyacyl-CoA dehydrogenase trifunctional multienzyme complex subunit α (HADHA), was detected in DNs. Treatment with etomoxir completely inhibited high-glucose-induced NET priming, acetyl-CoA accumulation, and histone acetylation. Furthermore, carbon flux analysis using U-^13^C glucose revealed increased amounts of unlabeled acetyl-CoA in HNs, suggesting that fatty acids are alternative sources of acetyl-CoA in neutrophils under hyperglycemia. However, we did not identify the mechanism underlying the enhanced FAO in hyperglycemic neutrophils. A possible explanation could be fatty-acid-favoring metabolic reprogramming of immune cells in diabetes. Insulin resistance and hyperglycemia are causally related to metabolic reprogramming in immune cells and vice versa. As the metabolic status determines immune responses, most immune cells display metabolic reprogramming under hyperglycemic conditions. For example, peripheral blood mononuclear cells (PBMCs) exhibit distinct lipid metabolism in a hyperglycemic environment [26]. Although PBMCs preferentially utilize glycolysis for ATP generation, PBMCs isolated from diabetic patients utilize fatty acids to generate T_H_17 cytokines [26]. An increase in CPT1 and a decrease in carnitine-acylcarnitine translocase ensured the accumulation of the long-chain fatty carnitine, which supports the production of T_H_17 cytokines. Macrophages under high-glucose conditions display impaired glycolysis without mitochondrial ATP production [2], and fatty acids induce macrophage polarization toward the proinflammatory phenotype [27]. Furthermore, FAO promotes the production of proinflammatory cytokines in macrophages through the activation of NLRP3 inflammation [28]. Therefore, our results suggest that DNs use FAO to provide sufficient amount of acetyl-CoA for NET priming.

Although neutrophils possess a complex mitochondrial network, they hardly adapt their mitochondria for energetics [29]. Rather, they utilize mitochondria for specialized functions, such as differentiation, apoptosis, and chemotaxis [30,31]. We found that mitochondrial respiration was not involved in high-glucose-induced NET priming. Neutrophils exhibited a low basal OCR, and diabetic conditions did not induce considerable changes in the OCR. In addition, treatment with oligomycin did not affect the ECAR in neutrophils. Nevertheless, our findings suggest a pivotal role for mitochondria in fatty acid metabolism under high-glucose-induced NET formation. Inhibition of CPT1 completely inhibited NET priming, acetyl-CoA accumulation, and histone acetylation. These results suggest that the mitochondria play a central role in FAO, glycolysis, and histone acetylation in HNs. Of note, the hearts of patients with diabetes showed similar metabolic adaptations. Heart homogenates of STZ-injected rats exhibited defects in mitochondrial complex I, but the mitochondrial defects did not limit the increases in FAO in the heart, leading to lysine acetylation of mitochondrial proteins [32]. Neutrophils possess very low levels of mitochondrial respiratory complex I [33]; we believe that this characteristic could be responsible for metabolic adaptation in HNs.

We found that ACLY mediates the acetylation of histones from accumulated acetyl-CoA in HNs. ACLY, a nuclear-cytosolic enzyme that generates acetyl-CoA from citrate produced through the mitochondrial TCA cycle [14], is responsible for histone acetylation in various cell types [14,17,34]. In particular, ACLY activation increases nuclear acetyl-CoA accumulation, and the resultant histone acetylation drives the expression of inflammatory genes in macrophages and T_H_17 cells [18,19]. Moreover, ACLY controls the generation of nucleocytosolic acetyl-CoA, which results in epigenetic regulation of cytokine responses through histone acetylation in T_H_17 cells [19]. Under diabetic conditions, neutrophils exhibited increased expression of ACLY, and fluorescence microscopic analysis revealed an increased nuclear translocation of ACLY. Moreover, inhibition of ACLY completely inhibited acetyl-CoA accumulation, histone acetylation, and NET priming. Therefore, we hypothesized that acetyl-CoA produced in mitochondria is converted into citrate through the TCA cycle and that ACLY mediates the nuclear production of acetyl-CoA from citrate. In support of this hypothesis, neutrophils under diabetic conditions showed the increased mitochondrial potential and increased CS expression levels, whereas the citrate level per se remained unaltered. Recent studies have suggested that histone acetylation is involved in NET formation. Increased histone acetylation caused by the inhibition of histone deacetylase promotes NET formation in resting and activated neutrophils [15]. NETs found in the patients with systemic lupus erythematosus contain increased amounts of acetylated histones, and histone deacetylase inhibitors prime neutrophils for NET formation [16]. We also observed that neutrophils under diabetic conditions exhibited acetylation of multiple histone lysine residues and that a pan-inhibitor of HATs completely inhibited the high-glucose-induced NET priming. Moreover, the pharmacological inhibition of ACLY successfully reversed delayed wound healing in a murine model of diabetes. These results suggest a close relationship between metabolic reprogramming and effector functions in neutrophils in the context of diabetes.

Interestingly, UDNs and HNs exhibited robust IL-1β production in response to LPS stimulation. They showed that increased basal levels of IL-1β and LPS stimulation further enhanced the production of IL-1β than NNs, whereas CDNs did not show any significant changes in IL-1β production in response to LPS stimulation. As the inflammasome is responsible for IL-1β production in neutrophils [35], these results suggest that metabolic reprogramming might affect the inflammasome activation in neutrophils. Moreover, a recent study shows that inflammasome activation is required for NET formation [36], suggesting that metabolic reprogramming might mediate inflammasome activation in DNs.

Our study demonstrates that trained immunity mediates NET priming of neutrophils in the diabetic environment. Metabolic reprogramming and epigenetic modifications enable neutrophils to exert proinflammatory responses, contributing to compromised wound healing in diabetes. However, our study has several limitations. Although FAO is an additional metabolic pathway for NET priming in DNs, no tracing of lipid metabolism was performed in this study to assess lipid-related metabolites. In addition, the master regulator of trained immunity in DNs remains unclear. Nevertheless, we propose that trained immunity in DNs could be a potential therapeutic target for attenuating inflammatory complications of diabetes.

## Materials and Methods

### Neutrophil isolation

This study included patients diagnosed with diabetes at Kyungpook National University Hospital between December 2019 and January 2023. Patients and healthy men (aged > 18 years) were enrolled in this study, and all study participants provided informed consent in accordance with the Declaration of Helsinki. Neutrophils were isolated as described previously [37]. Neutrophil purity was determined using Wright–Giemsa staining. The purity was found to be consistently >95%. Neutrophils (5 × 10^6^ cells) were incubated in 1 ml of glucose-free RPMI medium (Gibco) supplemented with either normal glucose (5.5 mM; BioBasic Inc.) or high glucose (22 mM) for 4 h. The HbA1c levels in patients with diabetes were adjusted according to the blood glucose levels [12]. Briefly, the mean plasma glucose levels were assumed to be a simple linear function of HbA1c levels, represented by the equation: Expected glucose levels (mg/dl) = 28.7 × HbA1c − 46.7. The patients’ glucose concentrations were measured and then classified as either controlled or uncontrolled on the basis of a comparison with the expected glucose level.

### Quantification of NET formation and ROS generation

Neutrophils were incubated under either normal- or high-glucose conditions for 4 h and then stimulated with either vehicle (double-distilled water), LPS (10 μg/ml; Sigma-Aldrich), or PMA (1 μg/ml; Sigma-Aldrich) for 1 h. The NET formation was quantified using SYTOX Green (Thermo Fisher Scientific) as previously described [38]. The fluorescence intensity was measured using a fluorescence microplate reader (SpectraMax M2/e, Molecular Devices Corporation) at excitation and emission wavelengths of 503 and 523 nm, respectively. ROS generation was measured using a fluorescence probe, 2′7′-dichlorodihydrofluorescein diacetate (DCF-DA; Thermo Fisher Scientific). Neutrophils were stained with DCF-DA for 30 min, and fluorescence intensity was measured using a fluorescence microplate reader at excitation and emission wavelengths of 490 and 520 nm, respectively. To determine the effects of metabolic pathway inhibitors, neutrophils were treated with the indicated inhibitors for 1 h before stimulation as follows: 50 μM ritonavir (Tocris Bioscience), 100 μM fasentin (Tocris Bioscience); 100 μM 2-DG (Sigma-Aldrich), 100 μM DMF (Sigma-Aldrich); 10 mM 6-AN (Sigma-Aldrich), 100 μM UK5099 (Tocris Bioscience), 300 nM CPI-613 (Cayman Chemical Company), 10 μM DCA (Sigma-Aldrich), 10 μM FX11 (Merck Millipore), 60 μM etomoxir (Sigma-Aldrich), 10 μM BMS303141 (Tocris Bioscience), and 10 μM DPI (Sigma-Aldrich).

### Seahorse XF analysis

The mitochondrial respiration and glycolytic functions were measured using an XF-96e extracellular flux analyzer (Seahorse Bioscience) according to the manufacturer’s protocol. Briefly, neutrophils (2 × 10^5^ cells) were seeded in the XF assay medium and plated on XF-96e tissue culture plates precoated with Cell-Tak (Corning Inc.). The ECAR of neutrophils was measured using an XF medium containing RPMI medium supplemented with 1 mM Seahorse XF glutamine solution (Agilent Technologies), followed by sequentially injection of glucose (10 mM), oligomycin (2 μM; Sigma-Aldrich), and 2-DG (10 mM). The OCR of neutrophils was measured using an XF medium containing RPMI medium supplemented with 10 mM Seahorse XF glucose solution (Agilent Technologies), 1 mM Seahorse XF pyruvate solution (Agilent Technologies), and 1 mM Seahorse XF glutamine solution (Agilent Technologies), followed by the sequentially injection of oligomycin (1 μM), carbonyl cyanide-4-(trifluoromethoxy) phenylhydrazone (2 μM; Sigma-Aldrich), rotenone (1 μM; Sigma-Aldrich), and antimycin A (1 μM; Sigma-Aldrich). XF analysis was performed at intervals of 6 min over a 90-min cycle at 37 °C.

### Mitochondrial membrane potential assay

A mitochondrial membrane potential assay with neutrophils was conducted using a JC-10 mitochondrial membrane potential assay kit (Abcam). Neutrophils (1 × 10^6^ cells) were incubated in 1× JC-10 dye-loading solution for 30 min at room temperature, and the mitochondrial membrane potential was analyzed using a BD FACSCalibur flow cytometer (BD Biosciences) in the FL1 (JC-10 green fluorescence) and FL2 (JC-10 red fluorescence) channels.

### Western blotting

Neutrophils (5 × 10^7^ cells/ml) were suspended in 500 μl of 1× radioimmunoprecipitation assay lysis buffer (Bio Basics) supplemented with 0.2% Triton X-100 (Sigma-Aldrich), a protease inhibitor cocktail (Sigma-Aldrich), and a phosphate inhibitor cocktail (Sigma-Aldrich), 20 μM phenylmethylsulfonyl fluoride (Sigma-Aldrich), leupeptin (10 mg/ml; Sigma-Aldrich), and aprotinin (Sigma-Aldrich) for 30 min. The proteins were extracted via centrifugation at 18,000*g* for 30 min at 4 °C. Total proteins (60 μg) were resolved using SDS-polyacrylamide gel electrophoresis, transferred to polyvinylidene difluoride membranes, blocked with 5% skim milk, and probed with primary antibodies against GLUT1 (Cell Signaling Technology), GLUT3 (Abcam), GLUT4 (Cell Signaling Technology), ACLY (Lifespan Biosciences), PDH (Cell Signaling Technology), histone H3 (Cell Signaling Technology), histone H4 (Cell Signaling Technology), AcH3K9 (Cell Signaling Technology), AcH3K14 (Cell Signaling Technology), AcH3K27 (Cell Signaling Technology), AcH4K8 (Cell Signaling Technology), P300/CBP-associated factor (PCAF) (KAT2B; Cell Signaling Technology), retinoblastoma binding protein (RBAP) (HAT1; Cell Signaling Technology), and β-actin (Santa Cruz Biotechnology).

### Immunofluorescence

Neutrophils were seeded on an eight-well chamber slide (Lab-Tek) precoated with 0.01% poly-l-lysine (Sigma-Aldrich) and fixed with 4% paraformaldehyde (Biosesang). Cells were permeabilized with 0.1% Triton X-100 and blocked with 5% donkey serum (Sigma-Aldrich) for 30 min at 37 °C. Neutrophils were incubated with primary antibodies against citrullinated H3 (CitH3; Abcam) and myeloperoxidase (MPO; Abcam), followed by fluorescence-conjugated secondary antibodies. To evaluate the mitochondrial membrane potential, neutrophils were stained with MitoTracker Red CMXRos (Thermo Fisher Scientific). To evaluate acetylated histones, neutrophils were incubated with primary antibodies against AcH3K9, AcH3K14, AcH3K27, and AcH4K8, and to evaluate ACLY and PDH, neutrophils were incubated with primary antibodies against ACLY and PDH1 (α_1_ and α_2_ subunits), further incubated with fluorescence-conjugated secondary antibodies. Cells were counterstained with 4′,6-diamidino-2-phenylindole (DAPI; Thermo Fisher Scientific) and visualized by either immunofluorescence microscopy (IX83, Olympus Optical Co. Ltd.) or confocal microscopy (LMS800, Carl Zeiss Meditec AG).

### RNA sequencing analysis

Total RNA was extracted from neutrophils (10^8^ cells) using TRIzol reagent (Ambion). Samples were prepared using the TruSeq Stranded Total RNA Sample Preparation Kit (Illumina). Paired-end cDNA libraries were prepared for each sample, and sequencing was performed using a NovaSeq 6000 system (Illumina). Library construction and RNA sequencing were conducted at Macrogen Korea. Bulk RNA sequencing data were analyzed using available packages in R. Adapter sequences from sequenced short reads were trimmed using the flexbar. These short reads were aligned with the human genome with align function (Rsubread v2.8.1) using a reference index built from GRCh38. The expression level of transcripts as read counts was estimated using featureCounts function (Rsubread v2.8.1; GRCh38.gtf annotation with default parameters). Differential expression analysis was performed using edgeR (3.36.0) using log transformation and the trimmed mean of *M* value method for normalization. The transcripts were annotated using org.Hs.eg.db database (v3.14.0). Pathway enrichment analysis was conducted with enrichGO and enrichKEGG functions from the clusterProfiler package (v.4.2.2.) with “Biological Process” as GO annotation term as pathways for GO enrichment analysis. Gene set enrichment analysis with clusterProfiler was performed for KEGG pathway enrichment using gseKEGG. The gene set enrichment analysis results and enriched KEGG pathways were visualized using a gseasPlot (clusterProfiler) and pathview (v1.34.0).

### Validation of differentially expressed metabolic genes by quantitative reverse transcription PCR

Total RNA was reverse-transcribed using a QuantiNova cDNA synthesis kit (QIAGEN). Real-time reverse transcription PCR was performed using SYBR Green reagent (QIAGEN) on a StepOnePlus system (Applied Biosystems). Gene-specific amplification was confirmed by comparative cycle threshold (*C*_t_) analysis using the StepOne software. The average *C*_t_ values of the genes were normalized to the average *C*_t_ values of β-actin, and the relative Δ*C*_t_ was used to calculate fold change values between the groups. Real-time PCR primers (QIAGEN) used in this study are the following: *PGLS* (PPH10822A-200), *ACACA* (PPH02316A-200), acetyl-CoA acetyltransferase 1 (*ACAT1*; PPH01600A-200), *ACLY* (PPH00021A-200), *CPT1C* (PPH11204A-200), *CS* (PPH2005F-200), *FASN* (PPH01012B-200), *GPI* (PPH00897C-200), *HK1* (PPH02045A-200), *HK2* (PPH00983B-200), *HK3* (PPH19558A-200), *H6PD* (PPH14128A-200), *HADHA* (PPH10000B-200), *LDHA* (PPH02047H-200), *PFKL* (PPH02048A-200), *PDHA1* (PPH07040A-200), *PKM1/M2* (PPH02050C-200), ribose-5-phosphate isomerase A (*RPIA*; PPH10180A-200), and transketolase (*TKT*; PPH10428A-200).

### Metabolic flux analysis

Neutrophils were plated in RPMI medium supplemented with either 5.5 or 22 mM ^13^C-U-glucose (Sigma-Aldrich) for 4 h. Cells were lyophilized, dissolved in 0.1% formic acid, and subjected to high-performance liquid chromatography (LC) using a Nexera system (Shimadzu Co.). The gradient solvent system consisted of 0.1% formic acid with water and 0.1% formic acid with acetonitrile. The LC system was coupled to an LC-mass spectrometry-8060 triple-quadrupole mass spectrometer (Shimadzu). As internal standards, 2-morpholino ethanesulfonic acid and methionine sulfone were used. The metabolites (focusing on glycolysis, PPP, and TCA cycle) were quantified, and ^13^C mass isotopic distributions were determined. The changes of metabolites were presented as relative enrichment with respect to the concentration using metabolites from NNs as control.

### Acetyl-CoA accumulation assay

The concentration of acetyl-CoA in neutrophils was determined using the PicoProbe Acetyl-CoA Assay Kit (Abcam). The CoASH Quencher and Quencher Remover were added to the sample, and the background fluorescence generated by free CoASH and succinyl-CoA was corrected. The acetyl-CoA fluorescence was measured using a microplate reader (SpectraMax iX3, Molecular Devices) at excitation and emission wavelengths of 535 and 587 nm, respectively. The background fluorescence generated by free CoASH and other CoA conjugates was reduced from the overall fluorescence, and relative concentration of acetyl-CoA was calculated.

### Colocalization analysis

The colocalization of ACLY with the nucleus was detected using EzColocalization. The single cells were identified as regions of interest (ROIs) based on the differential interference contrast images. Reporter 1 was assigned to DNA labeled with DAPI (blue), and reporter 2 was assigned to ACLY (green). Alignments were performed using the default threshold algorithm, and heatmaps were generated to visualize the relative magnitudes of the reporter signals. The colocalization coefficient was determined by calculating Pearson’s correlation coefficient (PCC).

### Animal experiments

Animal experiments were approved by the Institutional Animal Care and Use Committee of Kyungpook National University. C57BL/6J (male, 8- to 9-week-old) mice were obtained from Central Lab Animal Inc. Mice were injected intraperitoneally with STZ (50 mg/kg; Sigma-Aldrich, dissolved in 0.1 M citrate buffer, pH 4.5). Fasting blood glucose levels were monitored on day 6 after STZ injection, and mice with fasting blood glucose levels of >300 mg/dl were included in this study. Excisional dorsal full-thickness skin wounds (6 mm in diameter) were induced in the center of each dorsal skin on day 7 after STZ injection, and mice were administered either vehicle or bempedoic acid (10 mg/kg; MedChemExpress, dissolved in corn oil) on every 2 d. The wound area ratio was quantified by calculating the percentage of wound area compared to initial wound area.

### Statistical analysis

Data are presented as means ± SEM for continuous variables and as the number (%) for categorical variables. Statistical data were analyzed using GraphPad Prism (version 7.0e; GraphPad Software). Comparisons between 2 groups were performed using either a 2-tailed Student’s *t* test (parametric) or Mann–Whitney test (nonparametric test). Statistical significance was set at *P* < 0.05.

## Data Availability

RNA sequencing data generated during this study were deposited to Sequence Read Archive database using the dataset identifier PRJNA847641 (https://trace.ncbi.nlm.nih.gov/Traces/study/?acc=PRJNA847641&o=acc_s%3Aa).

## Supplementary Materials

Supplementary 1 Figs. S1 to S6 Tables S1 and S2 Supplementary Graphical Abstract
