# Supplementary material for: Diabetes Primes Neutrophils for Neutrophil Extracellular Trap Formation through Trained Immunity
Source: Research (Wash D C). 2024 Apr 23;7:0365. doi: 10.34133/research.0365 (PMC11037460; doi:10.34133/research.0365)
Supplement: Supplementary 1 — Figs. S1 to S6 Tables S1 and S2 Supplementary Graphical Abstract [file research.0365.f1.zip › Supplementary Figure Legends.docx]

**Supplementary Figure Legends**

**Figure S1. Related to Figure 1. Metabolic modulation in diabetic neutrophils.**

(***A‒E***) RNAseq analysis of diabetic neutrophils (DNs, *n* = 5) and normal neutrophils (NNs, *n*=4). (***A***) Heatmap representation of differentially expressed metabolic genes in NNs and DNs. (***B, C***) Gene Ontology (GO) tree of related GO biological processes (GOBP) for significantly upregulated genes in CDNs (***B***) and UDNs (***C***) with respect to NNs. (***D, E***) KEGG enrichment plot with gene counts and p-values for related KEGG pathways. (***F***) qPCR validation of the enzymatic genes for transketolase (*TKT*), ribose 5-phosphate isomerase A (*RPIA*), and hydroxyacyl-CoA dehydrogenase (*HADHA*), acetyl-CoA acetyltransferase 1 (*ACAT1*), lactate dehydrogenase A (*LDHA*), and *LDHAL6A* in DNs and HNs. *n* = 3‒4 per group. All results are expressed as mean ± SEM. **P* < 0.05; ***P* < 0.01.

**Figure S2. Related to Figure 2. Metabolic modulation of neutrophils during high glucose-induced NET priming.** (***A, B***) Effects of osmosis on NET formation. Human neutrophils were incubated in RPMI supplemented with either 5.5 mM glucose, 5.5 mM glucose + 5.5 mM mannitol, 11 mM glucose, 5.5 mM glucose + 16.5 mM mannitol or 22 mM glucose for 4 h. NET formation (***A***) and ROS generation (***B***) in neutrophils were analyzed by SYTOX green staining and DCF-DA staining, respectively. *n* = 3 per group. Results are expressed as mean ± SEM. **P* < 0.05. (***C–H***) Up, Enrichment plot for the indicated metabolic pathway with an enrichment score and rank for the associated gene set. Down, Integration of the enriched gene set into the KEGG map for indicated metabolic pathways. (***I***) A qPCR validation of the enzymatic genes of metabolic pathways in NNs and HNs. *HK1*, hexokinase1; *HK2*, hexokinase2: *HADHA*, hydroxyacyl-CoA dehydrogenase; *RPIA*, ribose 5-Phosphate Isomerase A, *TKT*, transketolase; and housekeeping gene *ACTB*, actin. (***J***) Schematic depicting altered metabolic pathways in DNs, based on KEGG metabolic pathway mapping. Red indicates upregulated genes, blue indicates down regulated genes, and grey indicates genes with no changes. (***K***) A qPCR validation of the enzymatic genes of metabolic pathways in NNs and neutrophils exposed to 11 mM glucose. All results are expressed as mean ± SEM. **P* < 0.05; ***P* < 0.01.

**Figure S3. Related to Figure 3. High-glucose exposure enhances glycolysis in neutrophils** (***A***) GLUT expression in neutrophils. HNs showed an enhanced GLUT4 expression. The left panels show representative immunoblot images of GLUT expression in NNs and HNs. The right panels show bar graphs of GLUT expression in NNs and HNs. *n* = 3. (***B***) Glucose uptake by neutrophils. Neutrophils were incubated under either normal- or high-glucose conditions and then subjected to glucose assays at the indicated time points. HNs exhibited enhanced glucose uptake at *4 h*. (***C***) Plots of basal OCRs and ECARs in NNs and HNs. (***D***) The mitochondrial transmembrane potential of neutrophils. Left panels: representative immunofluorescence images of NNs and HNs. Right panels: bar graph of MitoTracker fluorescence in NNs and HNs. *n* = 3. (***E***) The effect of diphenyleneiodonium chloride (DPI) on high glucose-induced NET priming. *n* = 7. (***F***) Cytokine production from neutrophils. Extracellular cytokine levels were measured in supernatants of neutrophils and intracellular cytokine levels were measured in total cellular proteins of neutrophils. All results are expressed as mean ± SEM. **P* < 0.05; ***P* < 0.01.

**Figure S4. Related to Figure 4. Labeling of glucose metabolites.** Metabolic flux analysis in normal glucose-exposed neutrophils (NNs) and high glucose-exposed neutrophils (HNs). Neutrophils were incubated in a medium supplemented with U-^13^C glucose, and the fates of labeled carbon were traced using LC-MS. Labeling isotopomer patterns of metabolic intermediates from the canonical glycolysis pathway (glucose-6-P and fructose-6-P) (***A***), PPP (6-phosphogluconate, ribose-5-P, eyrothose-5-P, and sedoheptulose-7-P) (***B***), pyruvate metabolism (glyceraldehyde-3-P, pyruvate, alanine, and lactate) (***C***), tricarboxylic cycle (citrate, succinate, fumarate, malate, aspartate, and glutamate) (***D***)**,** and acetyl-CoA (***E***). *n* = 3‒5 for each group. Data are presented as relative metabolite abundance and expressed as mean ± SEM.

**Figure S5. Related to Figure 5. The expression of ACLY and histone acetylation in high glucose-exposed neutrophils.** Representative immunofluorescence microscopic images of ACLY (***A***) and PDH (***B***) in neutrophils. Neutrophils were incubated with either 5.5 mm of glucose (normal glucose) or 22 mm of glucose (high glucose) for *4 h* and then subjected to immunofluorescence staining. (***C***) Subcellular localization of PDH in neutrophils. Left panels, immunofluorescence images showing reporter 1 (blue, 4ʹ,6-diamidino-2-phenylindole [DAPI]) and reporter 2 (green, PDH); second panels, differential interference contrast (DIC) images of cell identification; third panels, heatmaps for DAPI; and right panels, heatmaps for PDH. Representative images from five independent experiments are shown. Right panels, the probability of the colocalization of PDH with the nucleus determined using Pearson’s correlation coefficient (PCC) metric in the EzColocalization analysis. Representative images from five independent experiments are shown. The bar graph shows the percentage of colocalization between the PDH and the nucleus (*n* = 5). (***D–G***) Representative immunofluorescence microscopic images of histone acetylation in neutrophils. Neutrophils were incubated in either 5.5 mm glucose (normal glucose) or 22 mm glucose (high glucose) in the presence or absence of inhibitors of metabolic pathways for *4 h*. Neutrophils were subjected to immunofluorescence staining for AcH3K9 (***D***), AcH3K14 (***E***), AcH3K27 (***F***), and AcH4K8 (***G***). CPI-163, an inhibitor of PDH; DCA, an inhibitor of pyruvate dehydrogenase kinase; BMS303141, an inhibitor of ACLY; etomoxir, an inhibitor of CPT-1. Representative images from more than three independent experiments are shown. All results are expressed as mean ± SEM. **P* < 0.05; ***P* < 0.01; ****P* < 0.001.

**Figure S6. Related to Figure 6. The effect of ACLY inhibitor on excisional wound healing in a murine model of diabetes.** (***A***) Cytokine production in neutrophils isolated from patients with diabetes. Cytokine levels were measured from supernatants of whole blood isolated from patients with diabetes. Neutrophils were isolated from patients with diabetes, and extracellular cytokine levels were measured in supernatants of neutrophils. Intracellular cytokine levels were measured in total cellular proteins of neutrophils. (***B***) Schematic representation of the excisional sound model in a murine model of diabetes. (***C***) Blood glucose levels (***D***) Representative images of skin wounds in WT, STZ-treated, and STZ/ACLY inhibitor-treated mice. All results are expressed as mean ± SEM. **P* < 0.05; ***P* < 0.01.

**Graphical Abstract**

**
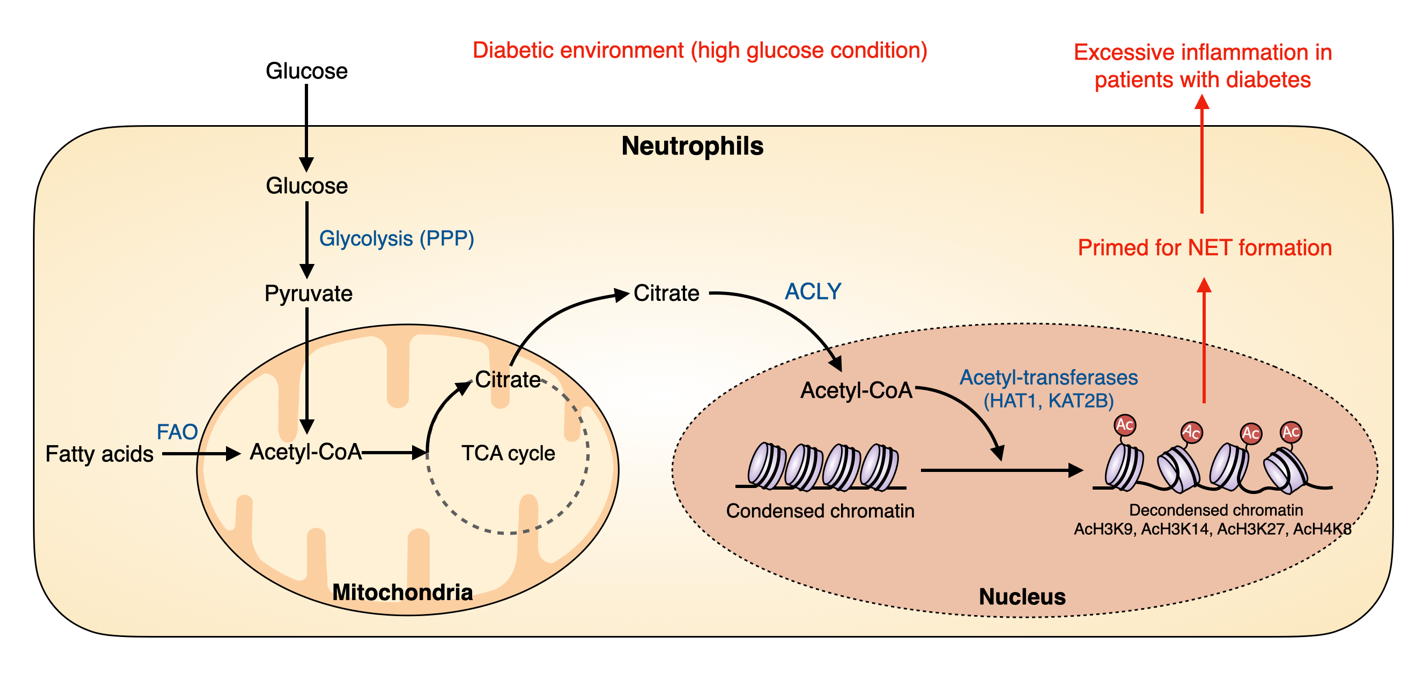
**

**Schematic representation of trained immunity in neutrophils in diabetes.** Neutrophils under diabetic conditions undergo metabolic reprogramming, comprising enhanced glycolysis via PPP and FAO. Nuclear localized ACLY-mediated accumulation of acetyl-CoA and HATs mediated the acetylation of lysine residues on histones 3 (AcH3K9, AcH3K14, and AcH3K27) and histone 4 (AcH4K4), which resulted in priming of neutrophils for NET formation.
