# Supplementary figures and images for: Diabetes Primes Neutrophils for Neutrophil Extracellular Trap Formation through Trained Immunity
Source: Research (Wash D C). 2024 Apr 23;7:0365. doi: 10.34133/research.0365 (PMC11037460; doi:10.34133/research.0365)

Figure S1 RNAseq analysis on diabetic neutrophils

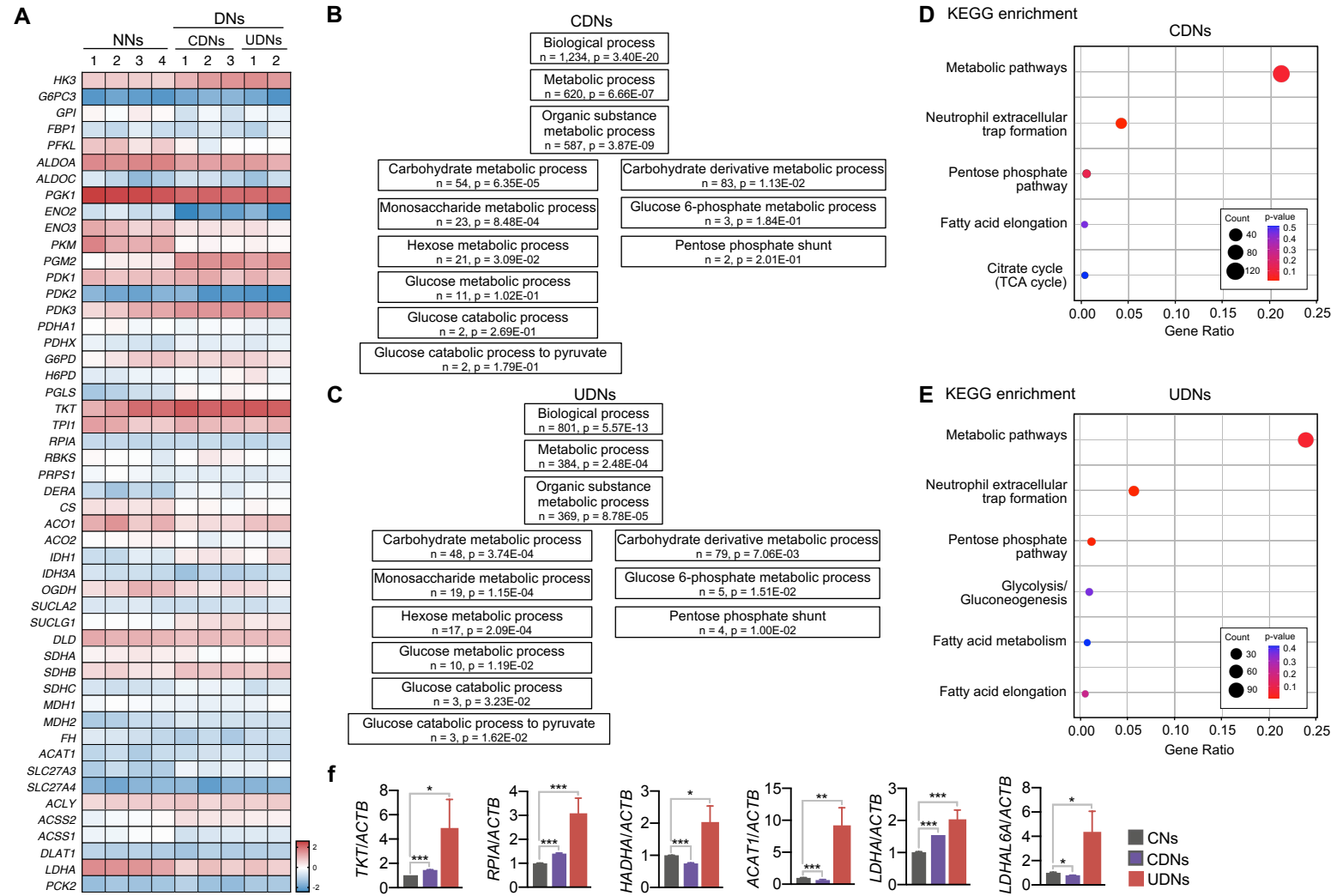

Supplement: Supplementary 1 — Figs. S1 to S6 Tables S1 and S2 Supplementary Graphical Abstract [file research.0365.f1.zip › FigureS01.pdf]

Figure S2

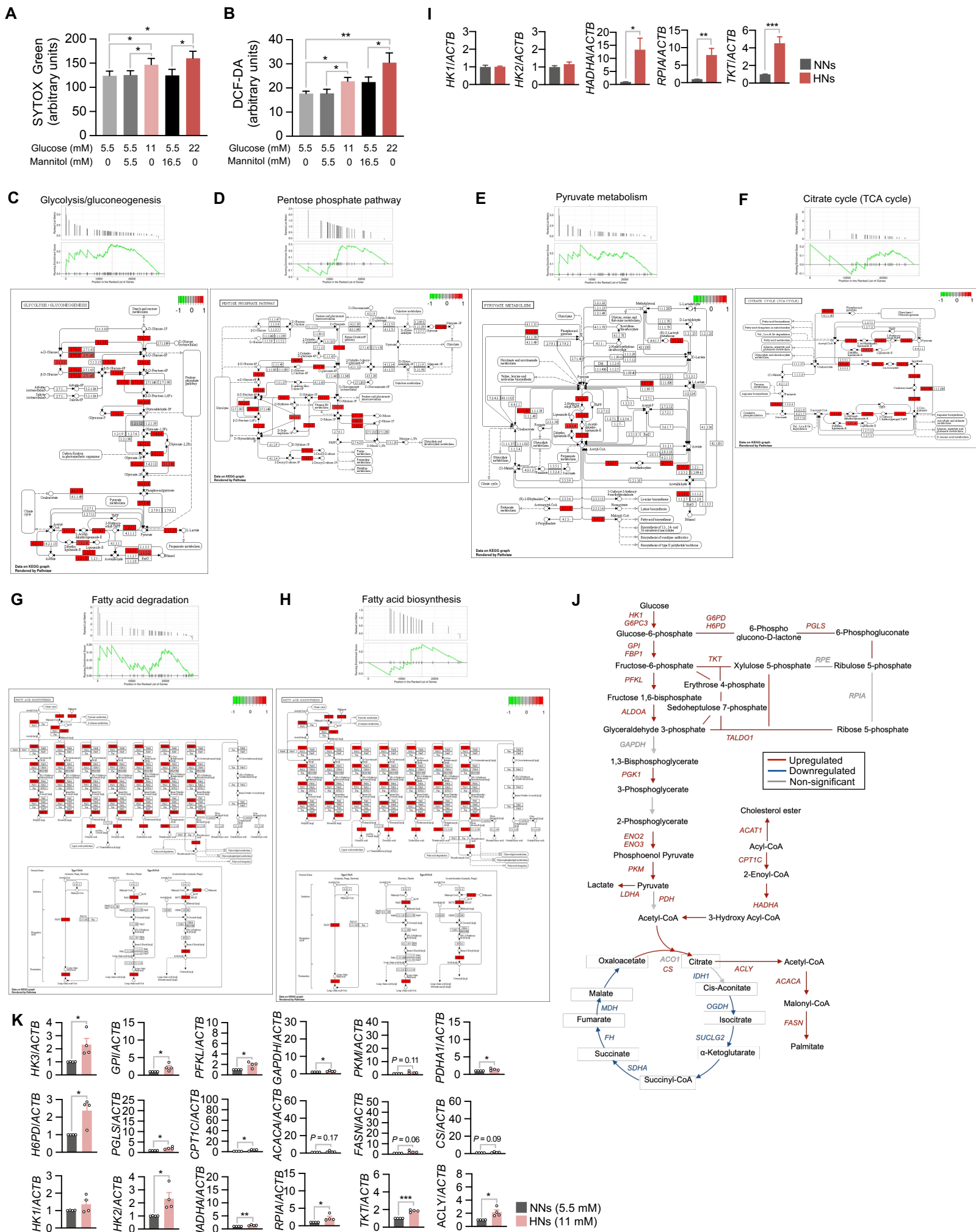

Supplement: Supplementary 1 — Figs. S1 to S6 Tables S1 and S2 Supplementary Graphical Abstract [file research.0365.f1.zip › FigureS02.pdf]

**Figure S3**

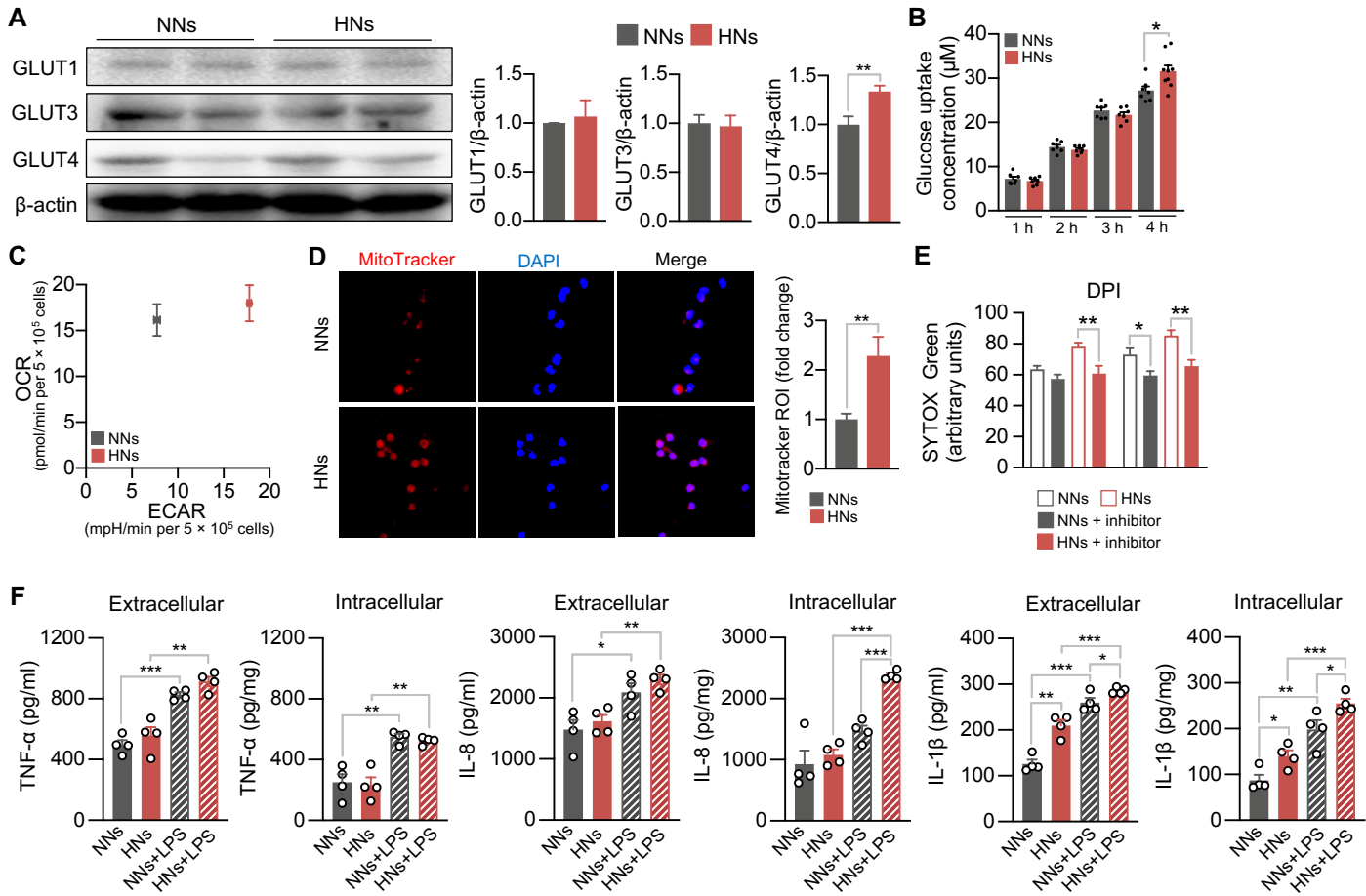

Supplement: Supplementary 1 — Figs. S1 to S6 Tables S1 and S2 Supplementary Graphical Abstract [file research.0365.f1.zip › FigureS03.pdf]

**Figure S4**

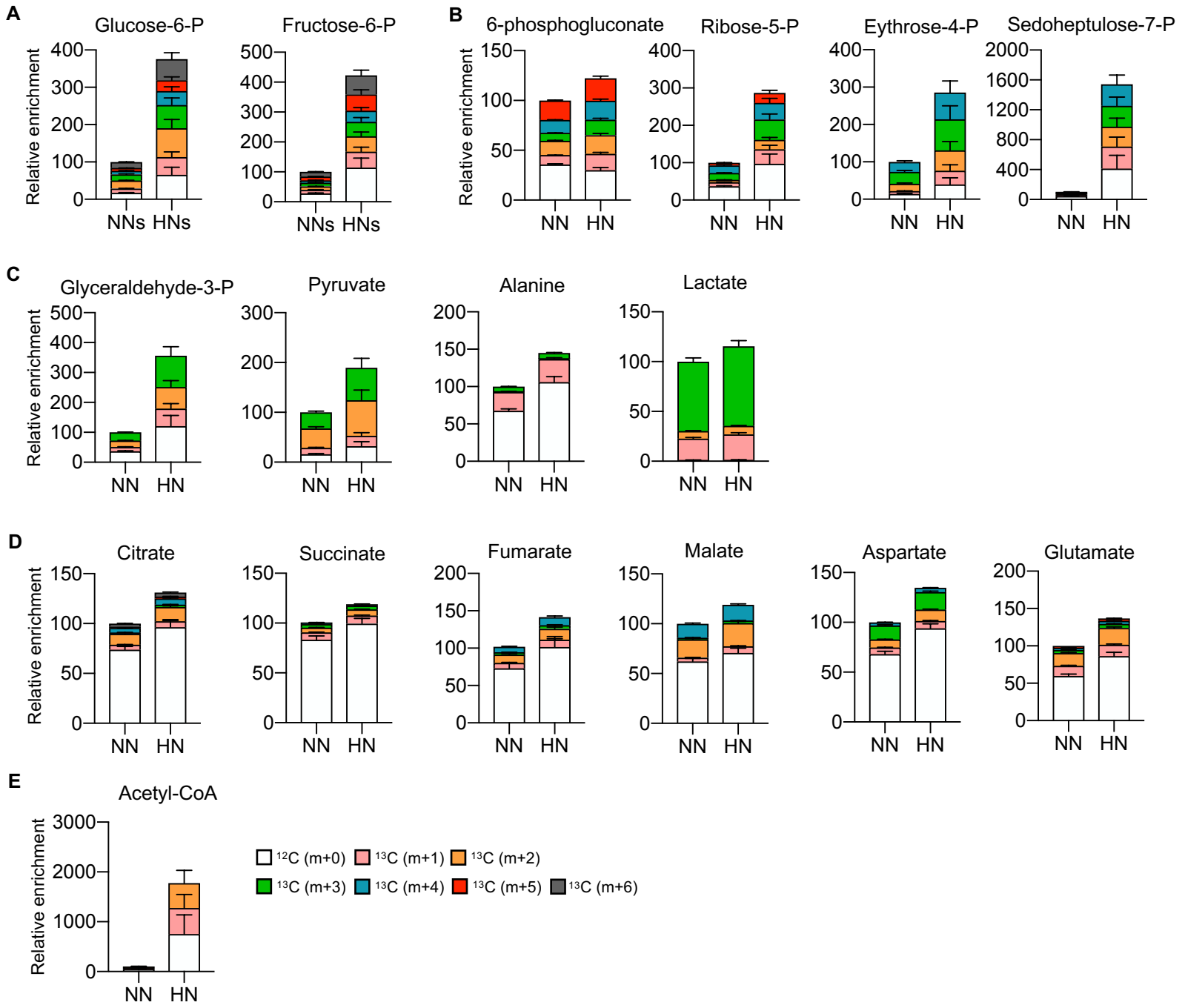

Supplement: Supplementary 1 — Figs. S1 to S6 Tables S1 and S2 Supplementary Graphical Abstract [file research.0365.f1.zip › FigureS04.pdf]

Figure S5

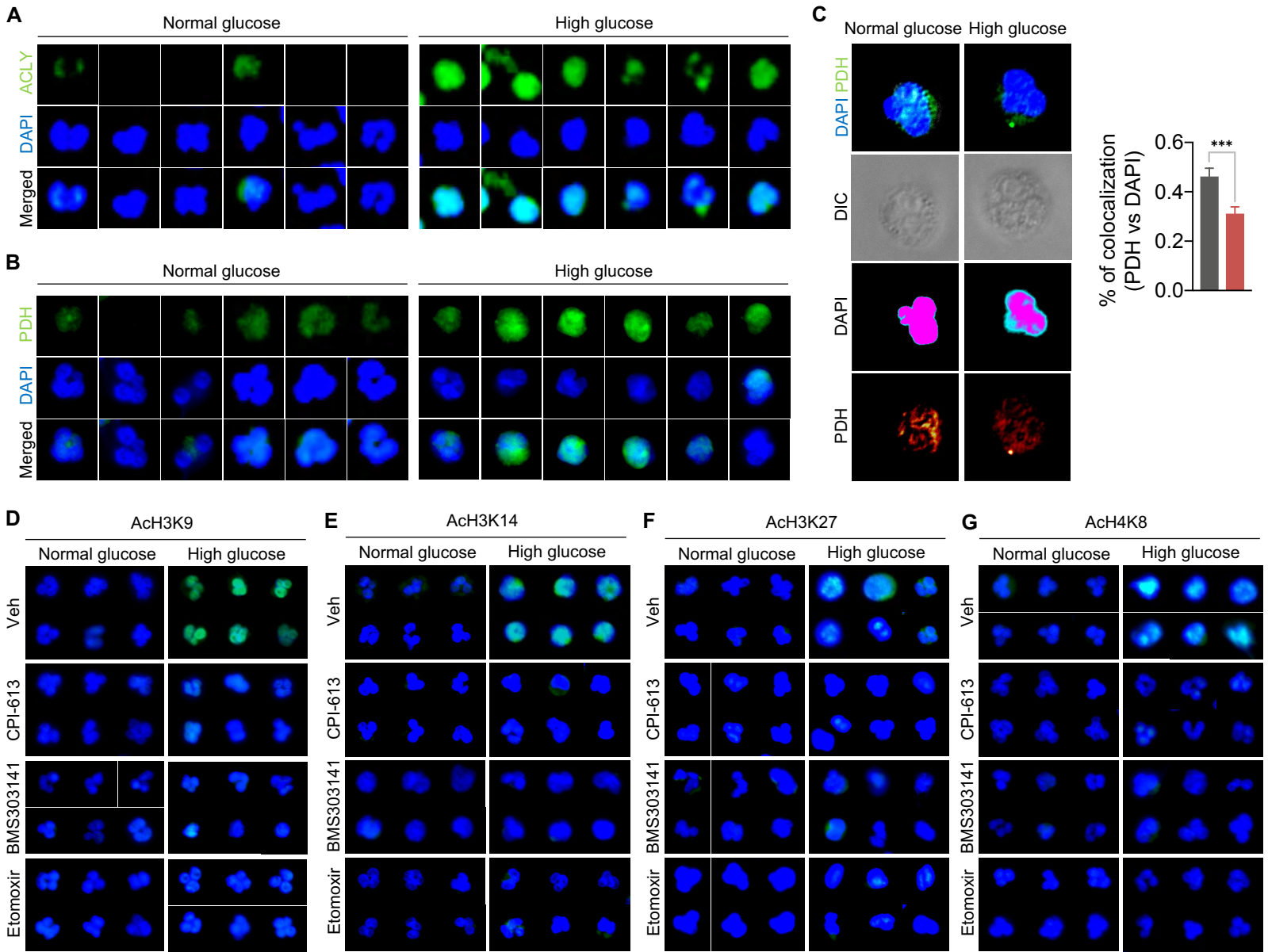

Supplement: Supplementary 1 — Figs. S1 to S6 Tables S1 and S2 Supplementary Graphical Abstract [file research.0365.f1.zip › FigureS05.pdf]

**Figure S6**

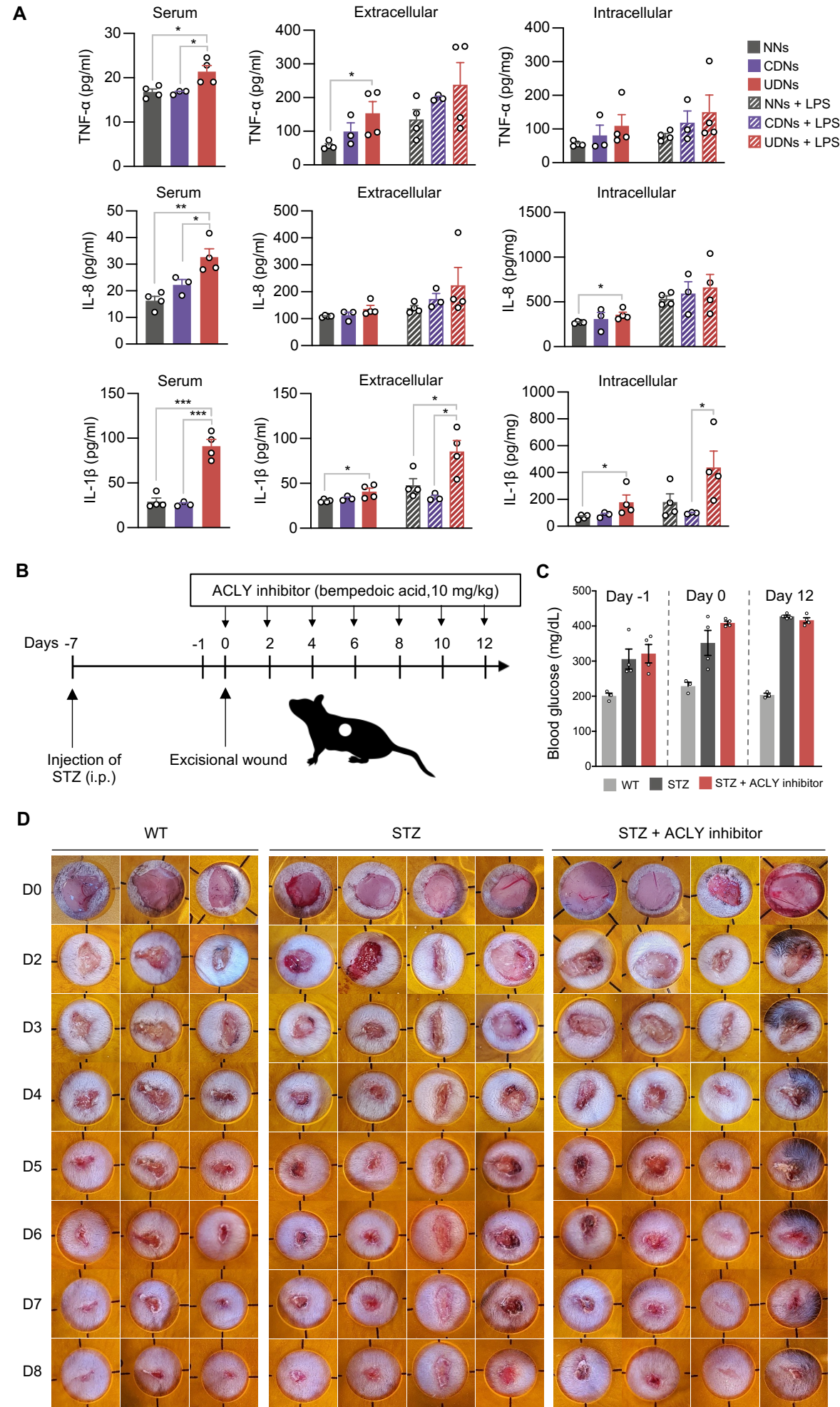

Supplement: Supplementary 1 — Figs. S1 to S6 Tables S1 and S2 Supplementary Graphical Abstract [file research.0365.f1.zip › FigureS06.pdf]

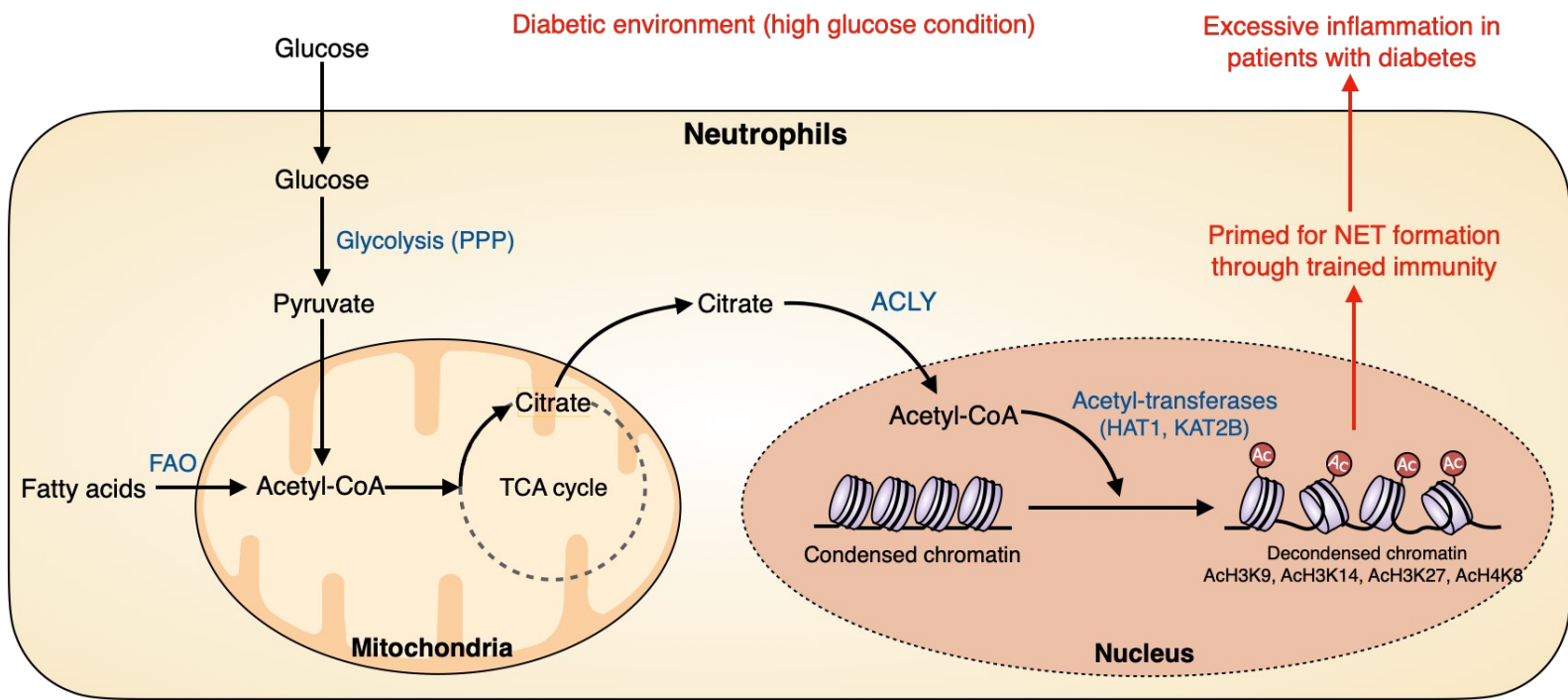

Supplement: Supplementary 1 — Figs. S1 to S6 Tables S1 and S2 Supplementary Graphical Abstract [file research.0365.f1.zip › Graphical_Abstract.pdf]
